# Gene loss and co-option of toll-like receptors facilitate paternal immunological adaptation in the brood pouch of pregnant male seahorses

**DOI:** 10.3389/fimmu.2023.1224698

**Published:** 2023-07-31

**Authors:** Bo Zhang, Wanghong Xiao, Geng Qin, Zelin Chen, Lihua Qiu, Xin Wang, Qiang Lin

**Affiliations:** ^1^ Key Laboratory of South China Sea Fishery Resources Exploitation & Utilization, Ministry of Agriculture and Rural Affairs, South China Sea Fisheries Research Institute, Chinese Academy of Fishery Sciences, Guangzhou, China; ^2^ Key Laboratory of Aquatic Product Processing, Ministry of Agriculture and Rural Affairs, South China Sea Fisheries Research Institute, Chinese Academy of Fishery Sciences, Guangzhou, China; ^3^ CAS Key Laboratory of Tropical Marine Bio-Resources and Ecology, South China Sea Institute of Oceanology, Chinese Academy of Sciences, Guangzhou, China; ^4^ Southern Marine Science and Engineering Guangdong Laboratory (Guangzhou), South China Sea Institute of Oceanology, Chinese Academy of Sciences, Guangzhou, China; ^5^ Sanya Institute of Ocean Eco-Environmental Engineering, Sanya, China

**Keywords:** seahorse, pregnancy, immune tolerance, host resistance, toll-like receptor

## Abstract

Male pregnancy in syngnathids (seahorses, pipefishes, and sea dragons) is an evolutionary innovation in the animal kingdom. Paternal immune resistance to the fetus is a critical challenge, particularly in seahorses with fully enclosed brood pouches and sophisticated placentas. In this study, comparative genomic analysis revealed that all syngnathid species lost three vertebrate-conserved *Toll-like receptors* (*TLR1, TLR2*, and *TLR9*), of which all play essential roles in immune protection and immune tolerance in the uterus and placenta. Quantitative real-time PCR (qRT-PCR) analysis showed that the *TLR* paralog genes including *TLR18*, *TLR25*, and *TLR21* were highly expressed in the placenta inside the seahorse brood pouch and changed dynamically during the breeding cycle, suggesting the potentially important role of the *TLR*s during male pregnancy. Furthermore, the immune challenge test *in vitro* showed a remarkable expression response from all three *TLR* genes to specific pathogenic antigens, confirming their immune function in seahorse brood pouches. Notably, the altered antigen recognition spectrum of these genes appeared to functionally compensate in part for the lost TLRs, in contrast to that observed in other species. Therefore, we suggest that gene loss and co-option of *TLR*s may be a typical evolutionary strategy for facilitating paternal immunological adaptation during male pregnancy.

## 1 Introduction

Pregnancy is one of the most significant achievements in vertebrate evolution (1). Offspring survival rate is remarkably improved via pregnancy by providing an ideal environment for embryo development and protecting the embryos from adverse external conditions (1). Pregnant mothers usually develop adaptive immune functions in the uterus, which protect the fetus via different immune pathways (2). However, a fundamental problem in the evolution of pregnancy among organisms is that pregnant mothers must avoid non-self-embryo rejection (3). Thus, appropriate maternal–fetal immune tolerance adjustments are vital for a successful pregnancy in viviparous animals (4).

The Syngnathidae family comprises seahorses, pipefish, and sea dragons, which are well known for their unique male pregnancy (5, 6). Males of most syngnathid species have evolved a brood pouch that function similarly to the mammalian uterus (7, 8). Seahorse brood pouch exhibits the most sophisticated morphological structures, a pocket-like structure in which eggs are embedded. The structures of seahorse brood pouch can be roughly divided into two layers: a folded inner pseudostratified columnar epithelium (termed placenta) and a smooth outer stratified cuboidal epithelium. The placenta serves as the site of embryo attachment (7, 9). During breeding cycle, the structure of the placenta exhibited a comparable physiological cycle to mammals uterus (9). It can be divided into three sequential cycle stages: the normal stage (non-pregnant stage), pregnant stage and the repair stage (10). The brood pouch not only provides shelter, nutrition, and immune protection for the embedded embryos, but also aids in avoiding rejection of the non-self-embryo from the pregnant father (8, 11–13). Previous studies have shown that the syngnathid immune system evolved via gene loss, mutation (14, 15), or expansion (13), thereby achieving a balance between immunological protection and embryo tolerance (10, 16). Thus, pregnant male syngnathids provide an excellent model for investigating the evolution of immunological adaptations during pregnancy (17).

The *Toll-like receptor* (*TLR*) gene family encodes pathogen recognition receptors (PRRs) of the immune system and plays vital roles in host immune responses (18, 19). As typical PRRs, TLRs discern invading microorganisms by recognizing pathogen-associated molecular patterns, leading to the activation of innate immune response or the development of antigen-specific acquired immunity (20, 21). Compared to that of other invertebrates, the *TLR* gene family in vertebrates evolved relatively conservatively (20). In addition, duplication, pseudogenization, loss, and positive selection of *TLR* members have been observed, particularly in species that have adapted to unique pathogenic environments (18, 22). *TLR*s within a subfamily usually recognize similar pathogen-associated molecular patterns (20). For example, members of the *TLR1* subfamily (including *TLR1, TLR2, TLR18*, and *TLR25*) have markedly similar ligand recognition profiles, including bacterial lipopolysaccharides (LPS), lipoteichoic acid (LTA), and peptidoglycan (PGN), etc. Although *TLR9* and *TLR21* belong to different subfamilies, both recognize bacterial and viral CpG-deoxynucleotides containing DNA (CpG-DNA) (23, 24). Therefore, compensatory effects are common among existing TLR members when certain TLRs lose their function (24).

TLR proteins are involved in immune activation and play important roles in mammalian pregnancy (25) by balancing host resistance and immune tolerance in the uterus and placenta during pregnancy (26, 27). Several *TLR*s such as *TLR1, TLR2*, and *TLR9* are expressed in placental immune cells (T cells and regulatory T (T_Reg_)), which directly participate in maternal-fetal immune tolerance during pregnancy (28, 29) and non-immune cells (such as trophoblasts and decidual cells) of the mammalian uterus and placenta (30). To guarantee conception and pregnancy, TLRs undergo remarkable expression changes (25, 31), which regulate maternal tolerance to allogenic fetuses and maintain innate immune responses to microorganisms (32).

To understand the potential function of the *TLR* gene family in paternal immunological adaptation in male pregnant syngnathids, we analyzed *TLR* families among syngnathids and other teleost species based on genome comparison. The expression patterns of *TLR*s with potential functions were detected in the placentas of seahorse brood pouches during the breeding cycle. Moreover, seahorse TLR ligands were recognized via an immune challenge *in vitro* to verify their immune function during pregnancy. Consequently, the immunological adaptive mechanisms caused by the *TLR* gene family evolution were successfully identified in syngnathids.

## 2 Results

### 2.1 TLR phylogenetic and comparative analyses

**Figure 1:**
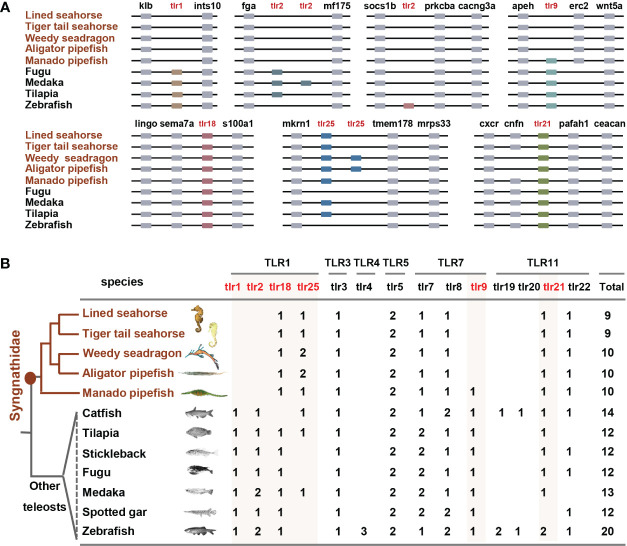
*TLR* gene family contracted in Syngnathidae fishes. **(A)** syntenic analysis plots show *TLR*s and their upstream and downstream genes, which are sequentially arranged and connected by black lines. Partial synteny map of the genomic region surrounding *TLR* family genes. **(B)** Statistical plot of *TLR* gene family in teleosts. *TLR1*, *TLR2* and *TLR9* were lost in Syngnathidae. Syngnathidae fishes and the focus *TLR*s are highlighted in red.

**Figure 2:**
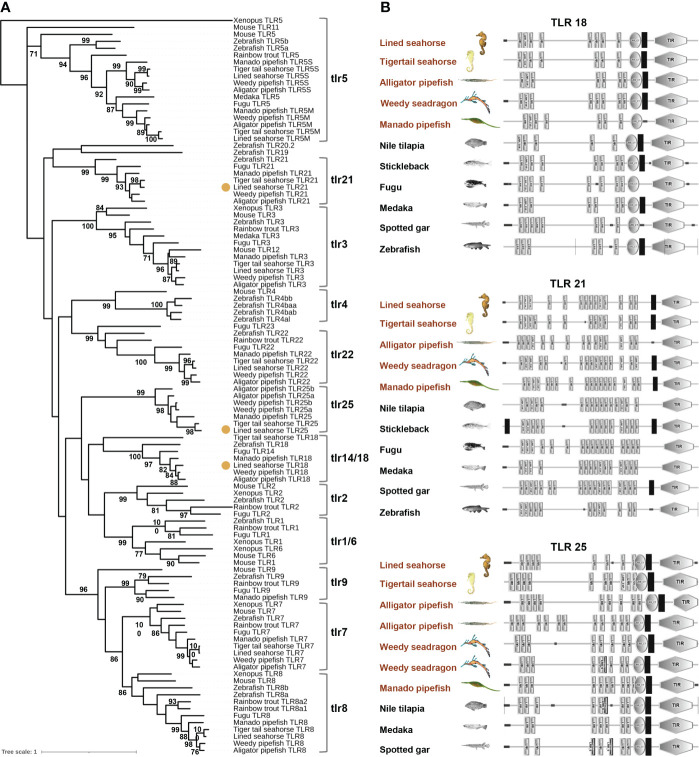
Phylogenetic analysis of teleost TLRs. **(A)** *TLR18*, *TLR21*, and *TLR25* of lined seahorse were note in colored dot. **(B)** Protein domain structures of *TLR18*, *TLR21* and *TLR25* in teleosts. LRR, leucine-rich repeat; LRR-TYP, leucine-rich repeat typical subfamily; TIR, Toll/IL-1 receptor; NT, N-(nitrogen) terminal; CT, C-(carboxyl) terminal.

Through genomic and transcriptome data analysis, *TLR* gene family members were identified in five Syngnathidae species: nine *TLR* genes were identified in lined and tiger tail seahorses, and 10 *TLR*s were identified in weedy, alligator, and manado pipefish (**Figure 1**). The phylogenetic tree divided Syngnathidae *TLR*s into five subfamilies: *TLR1, TLR3, TLR5, TLR7*, and *TLR11* (**Figure 2A**). Similar to most teleosts, Syngnathidae lack the *TLR4* subfamily. The *TLR1* subfamily comprises *TLR18* and *TLR25*, in addition to *TLR1, TLR2*, and *TLR14*. The *TLR3* and *TLR5* subfamilies include only one gene member each; namely *TLR3* and *TLR5*, respectively. *TLR7, TLR8*, and *TLR 9* formed the *TLR7* subfamily; *TLR21* and *TLR22* were clustered in a clade under the *TLR11* subfamily. Compared with that of other teleosts containing at least 12 *TLR* members, the *TLR* gene family was contracted in Syngnathidae fishes. In all five *Syngnathidae* fishes *TLR1* subfamily, *TLR1* and *TLR2* were lost except *TLR25*, which was present only in a few species. Notably, *TLR25* even showed an additional copy in weedy and alligator pipefish. Of the five Syngnathidae species, *TLR9* was absent in four excluding manado pipefish.

Further, *TLR* genes syntenic analyses revealed the different mechanisms of *TLR*s lost in Syngnathidae fish. The loss of *TLR1* in five Syngnathidae species (lined and tiger tail seahorses, weedy, alligator, and manado pipefish) was caused by a genomic fragment insertion. *TLR2* loss in the five studied Syngnathidae fish species was completely eliminated during genomic evolution. Although *TLR25* showed a tandom (localized) duplication at the adjacent locus in weedy and alligator pipefish, *TLR25* and *TLR21* showed conserved syntenies. Moreover, our results revealed different TLR constructions in the five Syngnathidae species. Similar to other vertebrate homologs, the domain architecture of these TLRs presented typical features of the TLR family, including a multiple-LRR domain at the N-terminal, central TM region, and Toll/IL-1 receptor (TIR) domain at the C-terminal (**Figure 2B**). However, the structures of the same *TLR* orthologs were different in different species, or even within the same species (**Figure 2B**). In general, *TLR* copy numbers and structures are variable, thus suggesting their potential for rapid mutation and possibility for the evolution of seahorse immune adaptation.

### 2.2 Expression profiles of *TLR18, TLR25* and *TLR21* in lined seahorse

**Figure 3:**
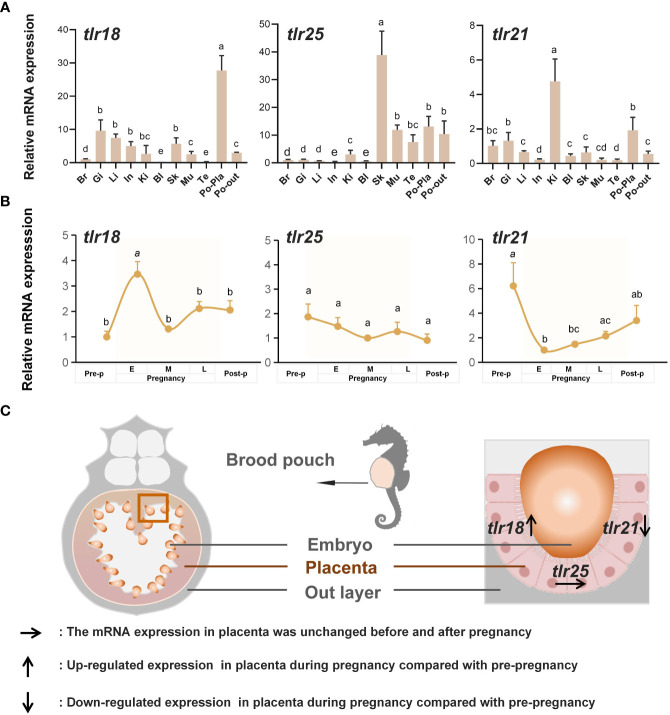
Temporal and spatial expression of seahorse TLRs. **(A)** *TLR18, TLR25*, and *TLR21* were highly expressed in placenta of seahorse brood pouch (mean ± SEM, *n* = 3). Br, brain; Gi, gill; Li, liver; In, intestine; Ki, kidney; Bl, blood; Sk, skin; Mu, muscle; Te, testis; Po-Pla, placenta of brood pouch; Po-out, out layer of brood pouch. Different letters indicate significant differences (*p* < 0.05). **(B)** The expression patterns of seahorse TLRs varied in the placenta during pregnancy cycle (mean ± SEM, *n* = 11). Different letters indicate significant differences (*p* < 0.05). **(C)** Schematic of expression pattern of TLRs in the seahorse placenta.

Due to the loss of conserved *TLR1* subfamily members *TLR1* and *TLR2* in Syngnathidae, we analyzed the spatiotemporal expression patterns of paralogs from the same subfamily (*TLR18* and *TLR25*). As *TLR9* is also absent in Syngnathidae, we examined the expression level of the seahorse *TLR21* gene, which is thought to recognize similar ligands, using RT-PCR (24). The expression profiles showed that *TLR18, TLR25*, and *TLR21* were ubiquitously expressed in all eleven examined tissues (brain, gill, liver, intestine, kidney, blood, skin, muscle, testis, placenta and out layer of brood pouch) in healthy lined seahorses and were highly expressed in immune and immune-related tissues (**Figure 3**). The highest *TLR18* expression was observed in the inner placenta of brood pouches. In addition, *TLR21* and *TLR25* were highly expressed in the placenta, with expression levels in the pouch second to the highest in tissues. At different breeding stages (pre-pregnancy, early-pregnancy, mid-pregnancy, late-pregnancy, and post-pregnancy), the expression patterns of *TLR18*, *TLR25*, and *TLR21* differed in the placenta; however, the expression of *TLR25* did not change significantly. *TLR18* was highly expressed in the early pregnancy stage and remained at a steady level during the subsequent stages. *TLR21* was initially highly expressed in the pre-pregnancy stages and subsequently decreased to a relatively low level, with a slight increase in the concluding stages. As well as, the genes expression of placenta T and T_reg_ cell surface marker molecules (33) significantly down-regulated at the mid- and later pregnancy stages (p<0.05) (**Supplementary Figure S2**). Taken together, all the three *TLR*s were highly expressed in the seahorse placenta, of which their expression patterns differed in the placenta during the pregnancy cycle. This suggested that the *TLR*s may function in placenta and their action modes may correspond to the placenta’s immunological demands at different breeding stages.

### 2.3 Ligand recognition profiles of TLR18, TLR25, and TLR21 in lined seahorse

**Figure 4:**
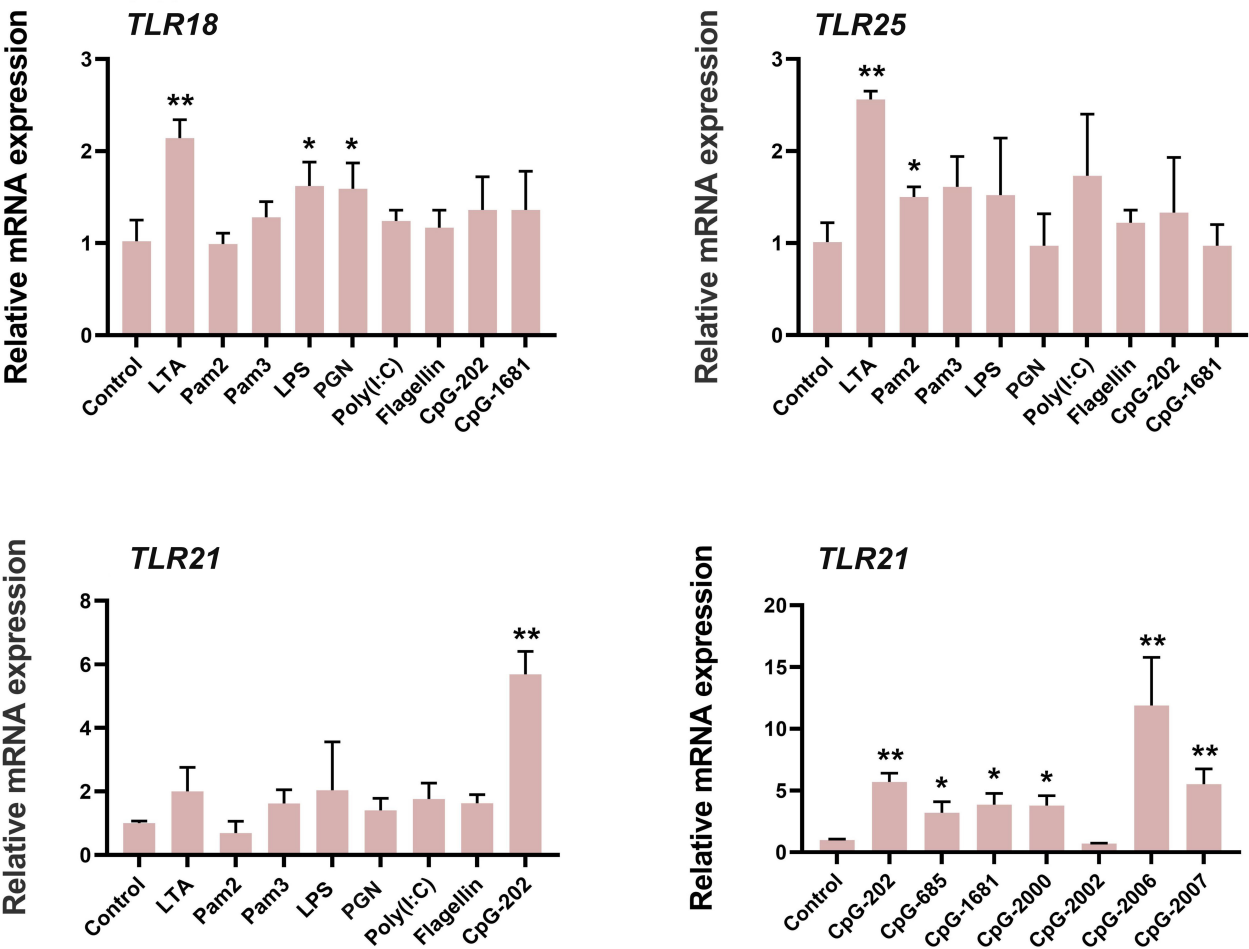
Ligand recognition profiles of TLR18, TLR25 and TLR21 in lined seahorse. Relative expression of *TLR* paralogues after stimulation with pathogen-associated molecular patterns are presented as the mean ± SEM (*n* = 5), and significant differences between control and treated groups are indicated with *(*p* < 0.05, Student’s t-test) or ** (*p* < 0.01, Student’s t-test).

We detected the ligand cognitive profiles of lined seahorse TLR18, TLR25, and TLR21 (**Figure 4**). The result showed that the expressions of *TLR18* and *TLR25* were significantly up-regulated after the LTA challenge. In addition, *TLR18* and *TLR25* expression were significantly upregulated after LPS and PGN stimulation, and synthetic diacylated lipopeptides (Pam2CSK4) stimulation, respectively. Seahorse *TLR21* was significantly upregulated after the CpG-ODNs challenge, particularly after the CpG-2006, CpG-202 and CpG-2007 challenges, which all contained the “GTCGTT” motif. In summary, these three TLRs responded to different antigens, indicating their involvement in seahorse immune defense. Different TLRs responded in different patterns, suggesting diversity in seahorse immune protection.

## 3 Discussion

Toll-like receptors (TLRs) are types of pathogen recognition receptors (PRRs) that play important roles in both vertebrate innate and acquired immunity (20, 34). The *TLR* gene family is an evolutionarily ancient family that has been widely retained in organisms ranging from cnidarians to humans (18). To date, 10 *TLR*s have been identified in humans (*TLR*1–*TLR10*), 12 in mice (*TLR1*–9 and *TLR11-13*), and 10 in birds (*TLR1a*, *TLR1b*, *TLR2a*, *TLR2b*, *TLR3*–*TLR5*, *TLR7*, *TLR15*, and *TLR21*) (18, 35). The repertoire of *TLR*s in fish is complex and varies among species owing to dynamic gene gain/loss during genome duplication events (36). In general, seven *TLR*s (*TLR1*-*3*, *TLR5* and *TLR7*-*9*) are orthologous to their mammalian and bird counterparts, which has been commonly conserved in teleosts (36). In addition, teleosts contain fish-specific *TLR*s: namely, *TLR14/18*–*27*. However, fish-specific *TLR*s vary among species (37). According to current research, the Atlantic cod (*Gadus morhua* L.) is the only known species lacking *TLR1*, *TLR2*, and *TLR5*, whereas an expansion of *TLR9* is present (five copies) (38). The unique *TLR* repertoires of fish are considered to be independent co-options during organismal evolution for adaption to specific immune demands (18). In the present study, we found that three conserved *TLR*s (*TLR1*, *TLR2*, and *TLR9*) were lost in syngnathids, which is unique among bony fish, thus indicating specific immune adaptation evolution in syngnathids. Male pregnancy in syngnathids is mainly carried out by the brood pouch, which is a unique trait in vertebrates (39). Similar to the mammalian uterus, the brood pouch provides a site for embryo implantation and is involved in both maternal immune tolerance and host resistance (8).

**Figure 5:**
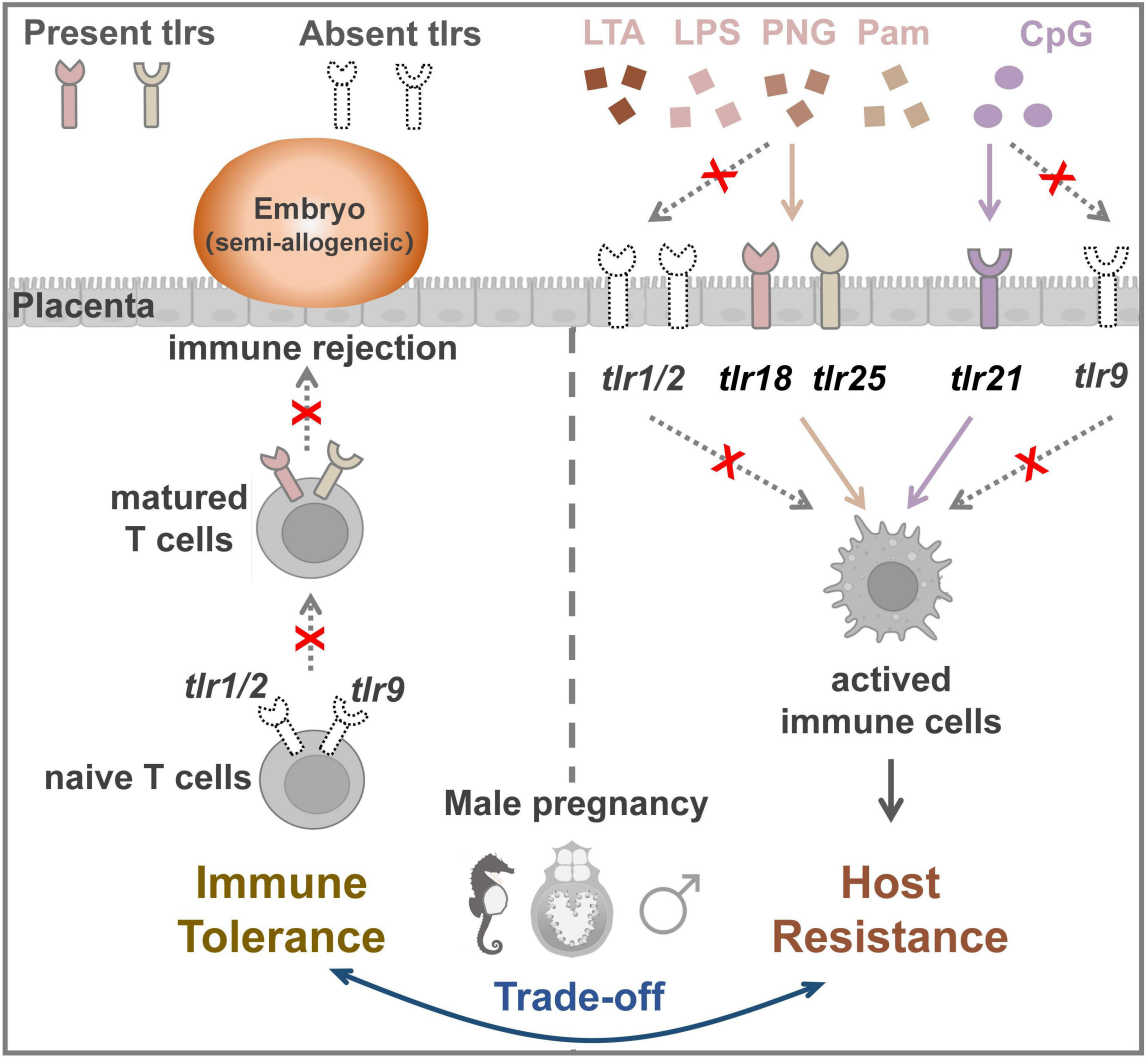
The co-option schedule of *TLR* adaptation to male pregnancy evolution. The loss of *TLR1*, *TLR2*, and *TLR9* might block immune rejection to facilitate the implantation of the embryo. The three *TLR*s (*TLR18*, *TLR25*, and *TLR21)* that were highly expressed in placenta partially and functionally compensated for the loss of the three conserved TLRs in host resistance.

To elucidate the immunological adaptive mechanism of syngnathid male pregnancy, two hypotheses have been proposed by different researchers based on current data. One hypothesis is that a trade-off between immunological tolerance and embryo rejection accompanies the evolution of unique male pregnancy, with the loss of several MHC II pathway genes in the pipefishes and a highly divergent invariant chain (CD74) in seahorses (14). The second hypothesis is that immunogenetic losses co-occurred with male seahorse pregnancy (5). In this study, we comprehensively analyzed the *TLR* gene family in teleosts and identified three conserved *TLR*s that were uniquely lost in pregnant male syngnathid fishes, suggesting that specific adaptive evolution commonly exists in syngnathids. Additionally, studies have confirmed that *TLR1*, *TLR2*, and *TLR9* are closely related to immune regulation during mammalian pregnancies (18, 40). The findings showed that *TLR1* variant ‘S248N’ influenced placental malaria during pregnancy (41) and that variants of *TLR2* were positively associated with recurrent pregnancy loss (42). *TLR9* activation coupled with IL-10 deficiency has been shown to induce adverse pregnancy outcomes (43). Kang et al. (44) found that excessive *TLR9* signaling contribute to the pathogenesis of spontaneous abortion by impairing T_reg_ cell survival via the activation of Caspase 8/3. Many studies have shown that activation of the TLR signaling pathway by exogenous and endogenous ligands can drive the induction of autoreactive T cells (28) and effector T cells to express *TLR*s (including but not limited to: *TLR1*, *TLR2*, and *TLR9*) (28, 45). The binding of TLR agonists to T cells contributes to their activation, which applies to both effector T cells and regulatory T (T_Reg_) cells (28). Maternal-fetal immune tolerance during pregnancy depends on the balance between effector T cells and T_Reg_ cells. T_Reg_ cells are among the most important cell types involved in establishing immune tolerance to self-antigens and antigens encountered in foreign grafts (29). In this study, the loss of three conserved *TLR*s (*TLR1*, *TLR2*, and *TLR9*) in syngnathids may influence the formation or maturation of T cells and T_Reg_ cells, which help to block paternal immune rejection. We hypothesized that the loss of *TLR1*, *TLR2*, and *TLR9* in syngnathids might be an adaptive evolution of paternal-fetal immune tolerance during pregnancy (**Figure 5**). Our findings provide new insight into the immune balance of male pregnancy in syngnathids.

In addition to their involvement in the adaptive immune regulation during pregnancy, TLRs are critical innate immune molecular defenses in mammalian uterine immune protection against infections by exogenous pathogens. By recognizing different types of pathogen-associated molecular patterns (PAMPs), TLRs activate downstream cascades as part of the innate immune response (20, 40). The expression of multiple *TLR*s in a temporal and spatial manner in the mammalian uterus has been reported in previous studies, which are believed to play an important role in the resistance of the uterus to pathogen infection and thus are necessary for embryo development (25, 30). In humans, *TLR1*, *TLR4*, *TLR7*, and *TLR8* have shown statistically significant increases in expression during the second trimester compared to that in the first trimester, which is believed to be conducive to pregnancy (25). Stimulation of *TLR2* and *TLR4* with zymosan and LPS induces IL-6 and IL-8 production in placental cultures, indicating that these placental TLRs can recognize pathogenic PAMPs and induce the innate immune responses (46). In the present study, seahorse *TLR18*, *TLR*25, and *TLR21* were highly expressed in the placenta and were varied. TLRs were significantly responsive to various PAMP agonists after stimulation. Thus, we concluded that these three TLRs play vital roles in the innate immune responses of seahorse brood pouches against infections by exogenous pathogens. Considering that the highest *TLR18* expression was observed in early pregnancy stages, this may indicate a strong immune response to eliminate pathogenic bacteria from the brood pouch, and thus provide a sterile environment for subsequent embryo development. When the brood pouch is open to the outside environment during non-pregnancy stages, the immune protection function mainly depends on *TLR21*; therefore, *TLR21* expression also increases.

Vertebrates exhibit immune redundancy or immune compensation effects. In zebrafish, both *TLR9* and *TLR21* recognize CpG-DNAs with partial functional redundancy (24). In chickens, the *TLR9* is lost from the genome and instead a functional institution by *TLR21* (23). To detect the potential immunological compensatory mechanism for the loss of the three conserved TLRs (*TLR1, TLR2*, and *TLR9*) in syngnathids, we examined the ligand recognition profiles of three other fish-specific *TLR*s (*TLR18*, *TLR21*, and *TLR25*) in the lined seahorse. We found that both *TLR18* and *TLR25* showed significant immune activation following the LTA challenge. In addition, *TLR18* showed an immune response to the LPS and PGN challenges, similar to *TLR25* in response to Pam2CSK4. *TLR21* showed an immune response only to CpG-DNAs, indicating strong immune specificity. Based on phylogenetic analyses, the vertebrate *TLR1*, *TLR2*, *TLR18*, and *TLR25* genes belonged to the *TLR1* subfamily and should therefore recognize the general class of associated PAMPs (20). In mammals, the bacterial cell-wall components the lipoproteins and lipopeptides are predominantly recognized by the TLR1 subfamily (47). TLR2 recognizes LTA, a characteristic component of the bacterial cell wall. In addition, TLR2 recognizes various other ligands from bacteria by forming heterodimers with TLR1 or TLR6. Synthetic synthetic triacylated lipopeptides (Pam3CSK4) is recognized by TLR2-TLR1 heterodimers, whereas Pam2CSK4 is a ligand for TLR2-TLR6 heterodimers (48). In European common carp (*Cyprinus carpio* L.), TLR2 can sense both PGN and LTA from Gram-positive *Staphylococcus aureus* and is less sensitive to the stimulation with Pam3CSK4 (49). In a study by Wei et al. (50), *TLR1* and *TLR2* expression in the spleen of the orange-spotted grouper (*Epinephelus coioide*) was up-regulated after LPS treatment. As found in our previous study (in which *TLR18* was mistermed as *TLR2*) (51) and in grass carp (*Ctenopharyngodon idella*), *TLR18* responds to LPS challenging. Our results supported the hypothesis that TLRs within a subfamily recognize a general class of associated PAMPs. Moreover, our study showed that TLR21 was significantly responsive to CpG-DNAs challenging, consistent with previous observations in zebrafish and chickens (23, 24). Thus, we suggest that the immune function of the lost vertebrate-conserved *LTR*s (*TLR1*, *TLR2*, and *TLR9*) is at least partially compensated for by *TLR18*, *TLR21*, and *TLR25* (**Figure 5**).

## 4 Materials and methods

### 4.1 Experimental animals and tissue sampling

Healthy lined seahorses (*Hippocampus erectus*) were collected from a seahorse breeding farm in Zhangzhou City, Fujian Province, China. All seahorses were kept temporarily in the seawater aquaculture system of the South China Sea Institute of the Chinese Academy of Sciences (Guangzhou, China) for two weeks. Seahorses were maintained in filtered circulating water under a 16:8 h light: dark cycle. Salinity, temperature, pH, and dissolved oxygen indices were 25 ± 1.0 ‰, 28 ± 0.5 °C, 7.5 ± 0.5, and 6.5 ± 0.5 mg·L^−1^ (mean ± SD), respectively.

For sample collection, seahorses were anesthetized with 0.05% tricaine methane sulfonate (MS222) before dissection. Eleven tissues (brain, gill, liver, intestine, kidney, gonad, muscle, skin, blood, and placenta and out layer of brood pouch) were surgically removed. According to the method of Zhang and Whittington (10, 52), the reproductive stages of brood pouch were judged by the placenta morphology and the embedded embryos development stage. In this study, five different reproductive stages (including pre-pregnancy, early-pregnancy, mid-pregnancy, late-pregnancy, and post-pregnancy) were divided. In brief, pre-pregnancy stage, no embryos embedded, thin and small blood vessels placenta; early-pregnancy stage, early-developed embryos (1-2 d) attached, vascularized with abundant blood vessels; mid-pregnancy stage, mid-developed embryos (8-12 d) attached, vascularized with abundant blood vessels; late-pregnancy stage, later-developed embryos (>15 d) attached, vascularized with abundant blood vessels; post-pregnancy stage, embryos released, blood vessels gradually resume the normal stage. To avoid RNA degradation, the collected samples were immediately frozen in liquid nitrogen and stored at −80°C. All experiments were conducted in accordance with the regulations of the Animal Research and Ethics Committee of the Chinese Academy of Sciences (approval number: SCSIO-IACUC-2019-000137).

### 4.2 Genome-wide identification of *TLR* genes in syngnathidae

Five representative fish of the Syngnathidae family were used for comparative *TLR* gene analysis. To identify the *TLR* genes, the TLR proteins of several representative vertebrates (including human (*Homo sapiens*), mouse (*Mus musculus*), chicken (*Gallus gallus*), channel catfish (*Ictalurus punctatus*), miiuy croaker (*Miichthys miiuy*), and zebrafish (*Danio rerio*)) were downloaded from the National Center for Biotechnology Information (NCBI) database as a query for native blast analysis in five Syngnathidae whole genomes and transcriptome databases (e-value ≤0.00001) (8, 10, 11, 53–55). To verify the *TLR* genes, the deduced TLRs’ architecture was analyzed using the SMART online prediction tool (http://smart.embl-heidelberg.de/) and sequences were identified using the NCBI Basic Local Alignment Search Tool (BLAST).

### 4.3 *TLR* genes evolution and compare

To elucidate the evolution of seahorse *TLR*s, phylogenetic analysis was performed based on 107 amino acid sequences obtained from 11 teleost fish species (including five Syngnathidae fish, five well-studied teleosts, and mice; see **Supplementary Table S1**). Multiple sequences were aligned using ClawstW and a phylogenetic tree was constructed using the maximum likelihood method in MEGA 6 with 1000 bootstrap replicates.

Syntenic analyses of *TLR*s were performed by comparing the locus position of *TLR* genes and upstream and downstream gene types. The genomes of five Syngnathidae fish species and other four representative teleosts (including zebrafish (*D. rerio*), tilapia (*Oreochromis niloticus*), fugu (*Takifugu rubripes*), and medaka (*Oryzias latipes*)) were utilized. Data for the five Syngnathidae and four representative teleosts were obtained from a previous study (55), and genomicus databases (http://www.genomicus.biologie.ens.fr/genomicus), respectively.

A genome-wide comparison of *TLR* construction in teleosts was conducted by comparing the genomic databases of five Syngnathidae fish with seven representative teleosts, including zebrafish (*D. rerio*), tilapia (*Oreochromis niloticus*), fugu (*Takifugu rubripes*), and medaka (*Oryzias latipes*), channel catfish (*Ictalurus punctatus*), spotted gar (*Lepisosteus oculatus*), and three-spined stickleback (*Gasterosteus aculeatus*)).

### 4.4 Tissue expression profile of *TLR18, TLR25* and *TLR21* in lined seahorse

Extracted seahorse tissues were ground in liquid nitrogen and total RNA was isolated using Trizol Reagent (Ambion, USA) according to manufacturer instructions (56). RNA was reverse transcribed into cDNA using the ReverTra Ace qPCR RT Master Mix kit (Toyobo, Japan) for different tissues. Gene expression across tissues for lined seahorse *TLR18*, *TLR21*, and *TLR25* was determined by quantitative real-time PCR (qRT-PCR). The primers (**Supplementary Table S2**) were designed using Primer 5.0 software (Plymouth, UK) and the specificity of the primers was detected by melting curve analysis. Primer amplification efficiency was evaluated using a standard curve (**Supplementary Figure S1**). The *β-actin* gene was used as an internal reference gene for relative quantification analysis. Amplification was carried out in Light Cycler 480 thermocycler (Roche, USA), and qRT-PCR was performed in 10 μl volumes (**Supplementary Table S3**) using SYBR Premix Ex-Taq™ reagent (Takara, Japan). The amplification reaction conditions and qRT-PCR parameters were as follows: denaturation for 3 min at 95°C; 40 cycles of 20 s at 95°C, 20 s at 58°C, and 20 s at 72°C; followed by 30 s at 95°C and 1 min at 60°C. C_T_ values were calculated with a fluorescence threshold of 0.5 to calculate relative gene expression. The relative mRNA expression of each gene was calculated using the 2^−ΔΔCt^ method (57). All data from the qRT-PCRs were presented as the mean ± standard error (SE). Statistical differences were estimated using unpaired Student’s t-tests or one-way ANOVAs followed by Tukey’s tests. All data in this study included three biological repeats in each group.

### 4.5 Expression profile of TLR18, TLR25, and TLR21 in seahorse breeding cycle

To detect whether specially evolved *TLR*s were involved in seahorse pregnancy, the expression profiles of *TLR18*, *TLR25*, and *TLR21* were detected in the placenta at five different reproductive stages (including pre-pregnancy, early-pregnancy, mid-pregnancy, late-pregnancy, and post-pregnancy). The detection was conducted by qRT-PCR as above.

### 4.6 Ligands recognition of seahorse TLR genes

To characterize the immune ligand recognition of TLR18, TLR21, and TLR25 in lined seahorses, eight typical TLR ligands (including 30 μg/mL LPS, 50 μg/mL LTA, 10 μg/mL Pam2CSK4, 10 μg/mL Pam3CSK4, 50 μg/mL polyinosinic:polycytidylic acid (Poly(I:C)), 10 μg/mL phosphorothioate-modified CpG-oligodeoxynucleotides (CpG-ODNs), 0.2 μg/mL Flagellin, and 50 μg/mL PGN) were selected to conduct a challenge experiment *in vitro*. The challenge experiment was conducted using a lined seahorse embryonic cell line (Chinese patent: ZL 2017 1 1050527.5) established in our on-site laboratory (52). Briefly, the cells were maintained in 6-well cell culture plates containing 5 mL Dulbecco’s modified eagle medium per well: Ham’s nutrient mixture F-12 (1:1) medium (DMEM/F12) supplemented with fetal bovine serum (FBS, 20%) at 28°C and 5% CO_2_ atmosphere. To address these challenges, cells were incubated with pathogenic ligands for 6 h and subsequently collected for TLR detection.

## Data availability statement

The original contributions presented in the study are included in the article/**Supplementary Material**. Further inquiries can be directed to the corresponding authors.

## Ethics statement

The animal study was reviewed and approved by the Animal Research and Ethics Committee of the Chinese Academy of Sciences (approval number: SCSIO-IACUC-2019-000137).

## Author contributions

QL and GQ supervised the project and designed the research. BZ and WX performed the genome and genetic analyses. BZ and WX performed qPCR & biological function detection. BZ, WX, ZC, LQ, and XW performed original draft writing and drawing figures. QL and GQ reviewed the writing. All authors contributed to the article and approved the submitted version.
