# Supplementary material for: Gene loss and co-option of toll-like receptors facilitate paternal immunological adaptation in the brood pouch of pregnant male seahorses
Source: Front Immunol. 2023 Jul 31;14:1224698. doi: 10.3389/fimmu.2023.1224698 (PMC10426278; doi:10.3389/fimmu.2023.1224698)
Supplement: Supplementary file 1 [file DataSheet_1.docx]

**Supplementary data**

**Gene loss and co-option of Toll-like receptor (TLR) facilitate paternal immunological adaptation in the brood pouch of male pregnant seahorse**

## Content:

**1 Supplementary Figures S1-2**

**2 Tables S1–3**


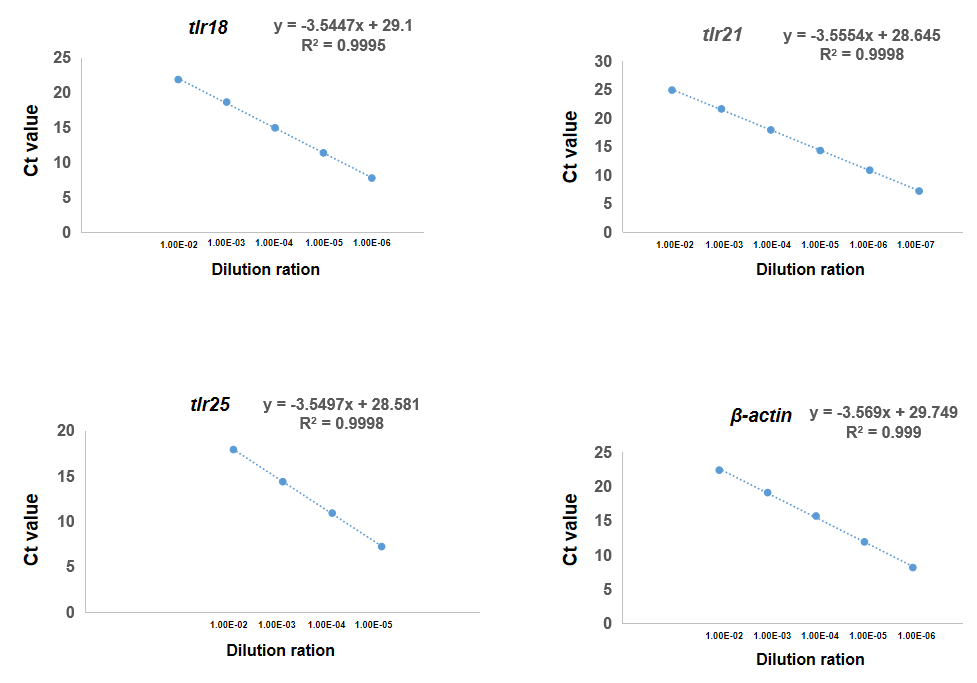
**Supplementary Figure S1**. Curves show values for the dilution ration of plasmid plotted against Ct values from qRT-PCR amplification.


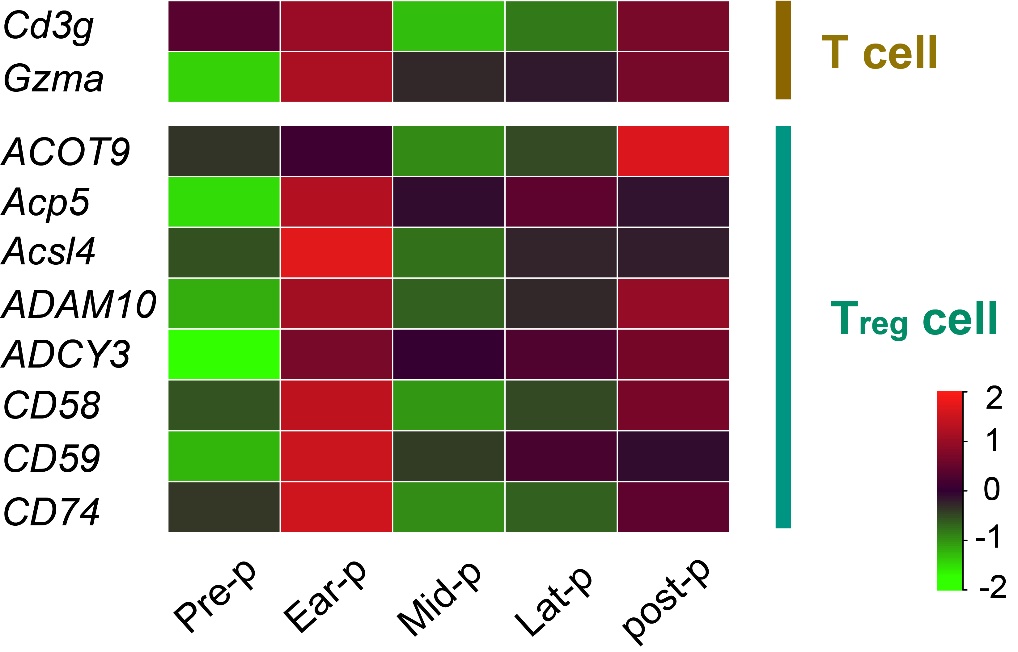


**Supplementary Figure S2**. The gene expression profiles of T cell and T_reg_ surface marker genes during seahorse pregnancy cycle. The Cd3g and Gzma is the placenta T cell surface marker molecules. The rest were cell surface marker molecules of T_reg_ cells.

**2 Tables S1–3**

Table S1. Gene IDs of TLR sequences used for phylogenetic trees

| Species | Gene name and IDs |
| --- | --- |
| Lined seahorse  (*Hippocampus erectus*) | tlr3：EVM0009521 |
|  | tlr5M：EVM0016828 |
|  | tlr5：EVM0014216 |
|  | tlr7：EVM0004238 |
|  | tlr8：EVM0016819 |
|  | tlr18：EVM0002937 |
|  | tlr21: EVM0001806 |
|  | tlr22: EVM0009244 |
|  | tlr25: EVM0004890 |
| Tiger tail seahorse  (*Hippocampus comes*) | tlr3: XM_019862645 |
|  | tlr5M: XM_019896224 |
|  | tlr5S: XM_019890715 |
|  | tlr7: XM_019895543 |
|  | tlr8: XM_019895544 |
|  | tlr18: XM_019863767 |
|  | tlr21: XM_019896043 |
|  | tlr22: XM_019884774 |
|  | tlr25: XM_019866170 |
| Weedy pipefish  (*Phyllopteryx taeniolatus*) | tlr3: EVM0002544.1 |
|  | tlr5M: EVM0010121.1 |
|  | tlr5: EVM0011220.1 |
|  | tlr7: EVM0016618.1 |
|  | tlr8: EVM0006783.1 |
|  | tlr18: EVM0008194.1 |
|  | tlr21: EVM0007128.1 |
|  | tlr22: EVM0005841.1 |
|  | tlr25: EVM0008620.1 |
|  | tlr25: EVM0009363.1 |
| Manado pipefish  (*Microphis manadensis* ) | tlr3: EVM16765 |
|  | tlr5M: EVM12008 |
|  | tlr5: EVM12059 |
|  | tlr7: EVM13091 |
|  | tlr8: EVM13092 |
|  | Tlr9: EVM02350 |
|  | tlr18: EVM16641 |
|  | tlr21: EVM18350 |
|  | tlr22: EVM17282 |
|  | tlr25: EVM02613 |
| Alligator pipefish  (*Syngnathoides biaculeatus*) | tlr3: EVM0004540.1 |
|  | tlr5M: EVM0014710.3 |
|  | tlr5: EVM0004827.1 |
|  | tlr7: EVM0020298.1 |
|  | tlr8: EVM0001797.1 |
|  | tlr18: EVM0019716.1 |
|  | tlr21: EVM0006499.1 |
|  | tlr22: EVM0007489.1 |
|  | tlr25: EVM0005977.1 |
|  | tlr25: EVM0007839.1 |
| Torafugu  (*Takifugu rubripes*) | tlr1: XM_003970363.3 |
|  | tlr2: KY774385.1 |
|  | tlr3: AC156436.1 |
|  | tlr5: AC156437.1 |
|  | tlr7: KY774386.1 |
|  | tlr8: AC156438.1 |
|  | tlr9: AC156439.1 |
|  | tlr14: AC156431.1 |
|  | tlr21: KY774387.1 |
|  | tlr22: AC156434.1 |
|  | tlr23: AC156435.1 |
| Japanese medaka  (*Oryzias latipes*) | tlr3: XM_004066015.4 |
|  | tlr5: XM_024291990.1 |
| *Rainbow trout*  (*Oncorhynchus mykiss*) | tlr1: GQ502185.1 |
|  | tlr2: XM_021578334.1 |
|  | tlr3: NM_001124578.1 |
|  | tlr5: AB062504.1 |
|  | tlr7: GQ422119.1 |
|  | tlr8: GQ422121.1 |
|  | tlr9: EU627195.1 |
|  | tlr22: NM_001124412.1 |
| Tropical clawed frog  (*Xenopus tropicalis*) | tlr1: XM_018095354.2 |
|  | tlr2: XM_004911150.4 |
|  | tlr3: XM_002934402.4 |
|  | tlr5: NM_001078891.1 |
|  | tlr6: XM_018095356.2 |
|  | tlr7: NM_001127411.1 |
|  | tlr8: XM_002933813.2 |
| Zebrafish  (*Danio rerio*) | tlr1: NM_001130593.1 |
|  | tlr2: NM_212812.1 |
|  | tlr3: NM_001013269.3 |
|  | tlr4a1: NM_001328605.1 |
|  | tlr4ba1: KF582561.1 |
|  | tlr4ba2: NM_001131051.1 |
|  | tlr4bb1: KF582562.1 |
|  | tlr4bb2: NM_212813.2 |
|  | tlr5M: MF983798.1 |
|  | tlr5S: MF983797.1 |
|  | tlr7: XM_021479060.1 |
|  | tlr8a: XM_001920559.6 |
|  | tlr8b: XM_017358120.2 |
|  | tlr9: NM_001130594.1 |
|  | tlr18: NM_001089350.1 |
|  | tlr19: NM_001365424.1 |
|  | tlr20: NM_001177443.2 |
|  | tlr21: NM_001199335.1 |
|  | tlr22: NM_001128675.2 |
| *House mouse* (*Mus musculus*) | tlr1: AY009154.1 |
|  | tlr2: AF185284.1 |
|  | tlr3: AF355152.1 |
|  | tlr4: AF177767.1 |
|  | tlr5: AF186107 |
|  | tlr6: AF314636.1 |
|  | tlr7: AY035889.1 |
|  | tlr8: AY035890.1 |
|  | tlr9: AF348140.1 |
|  | tlr11: AY510704.1 |
|  | tlr12: AY510705.1 |
|  | tlr13: AY510706.1 |

Table S2. Primer sequences of seahorse TLRs used for quantitative real-time PCR.

| Specie | Gene name | Primer sequences (forward and reverse; 5′→ 3′) |
| --- | --- | --- |
| Lined seahorse | Tlr18-F | TGACCAACCTACAAGTCGCC |
|  | Tlr18-R | GTCGCAAGGGACAGAGTTGA |
|  | tlr21-F | CTTCGCCTGCTTCGGTTAGACAA |
|  | tlr21-R | GCTCAAGTTGGACACGGAACGG |
|  | tlr25-F | TCGTACAGTCACAAGGACGC |
|  | tlr25-R | GGATGGGTTCCAACAGGAGG |

### Table S3. qPCR amplification system

| Composition of reaction | Volume (μL) |
| --- | --- |
| H_2_O | 3 |
| SYBR Green Real-time PCR | 5 |
| Sense primer (10 μM) | 0.5 |
| Antisense primer (10 μM) | 0.5 |
| cDNA | 1 |
| Total volume | 10 |
